# Supplementary material for: Normalizing HIF-1α Signaling Improves Cellular Glucose Metabolism and Blocks the Pathological Pathways of Hyperglycemic Damage
Source: Biomedicines. 2021 Sep 2;9(9):1139. doi: 10.3390/biomedicines9091139 (PMC8471680; doi:10.3390/biomedicines9091139)
Supplement: Supplementary file 1 [file biomedicines-09-01139-s001.zip › biomedicines-1338225-supplementary.pdf]

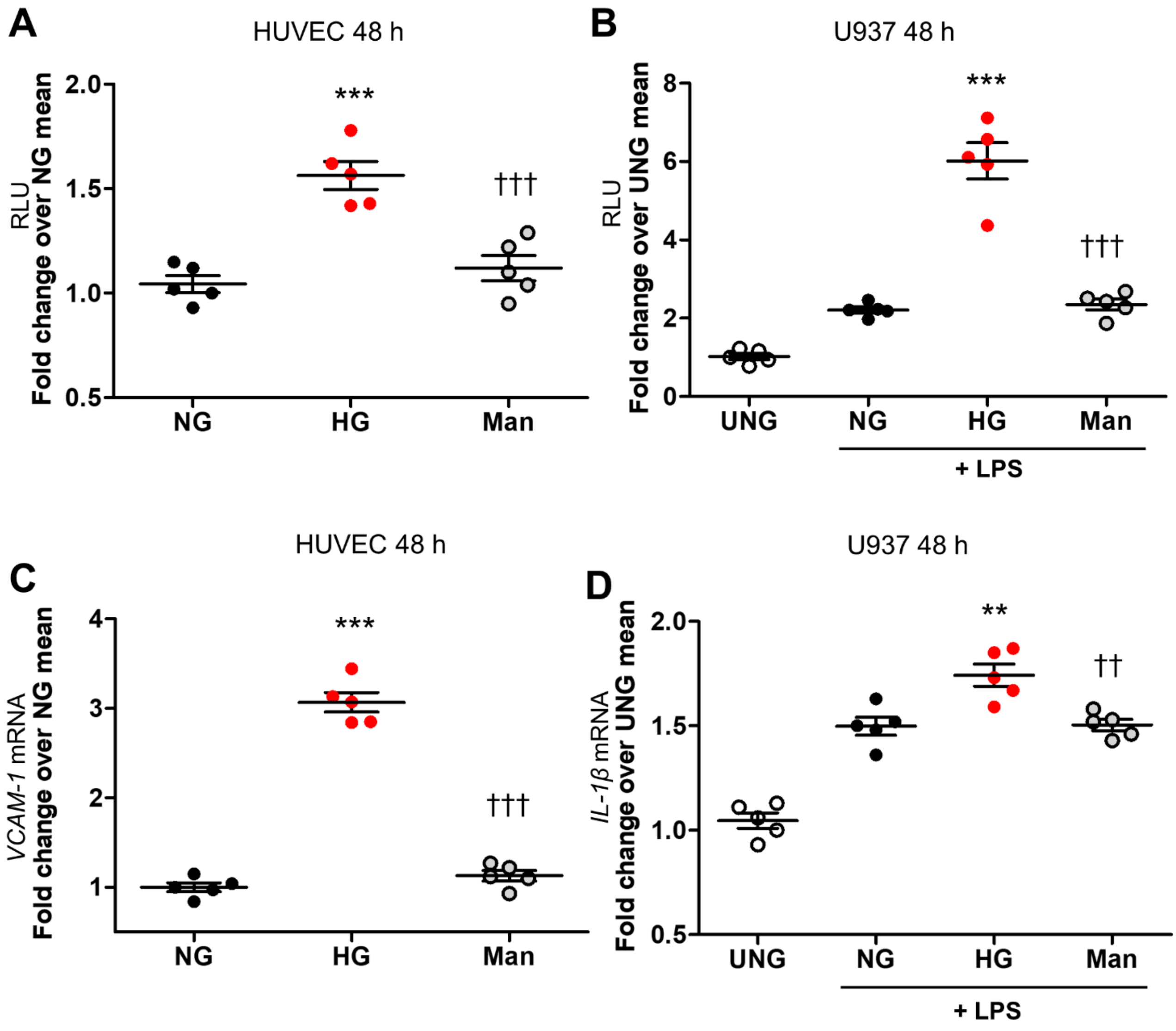

Supplementary Figure S1. Mannitol 20 mM does not induce HIF-1α activity and proinflammatory activation of endothelial cells and LPS-stimulated macrophages. HIF-1 activity, as assessed by dual-luciferase gene reporter assay, in HUVEC (**A**), and in U937 macrophages stimulated (or not, UNG = untreated normal glucose) with LPS (10 ng/mL) (**B**), after 48 h incubation with high glucose (HG, 20 mM) or mannitol (Man, 20 mM) vs NG (5.5 mM); n = 5 wells in duplicate per condition. *VCAM-1* (**C**) mRNA levels in HUVEC, and *IL-1β* mRNA levels in U937 macrophages stimulated (or not, UNG) with LPS (**D**), exposed to HG or Man 20 mM vs NG for 48 h.; n = 5 wells in duplicate per condition. Each dot represents the mean of two individual technical replicate and bars represent mean ± SEM. Post hoc multiple comparison: ***P < 0.001 or **P < 0.01 vs NG; †††P < 0.001 or †P < 0.01 vs HG.
